# Consolidating strategic planning and operational frameworks for integrated vector management in Eritrea

**DOI:** 10.1186/s12936-015-1022-7

**Published:** 2015-12-02

**Authors:** Emmanuel Chanda, Birkinesh Ameneshewa, Selam Mihreteab, Araia Berhane, Assefash Zehaie, Yohannes Ghebrat, Abdulmumini Usman

**Affiliations:** Vector Control Specialist/Consultant, Kamwala South, Lusaka, Zambia; World Health Organization, Regional Office for Africa, Brazzaville, Congo; National Malaria Control Programme, Ministry of Health, Asmara, Eritrea; Communicable Diseases and Control Division, Ministry of Health, Asmara, Eritrea; World Health Organization, Eritrea Country Office, Asmara, Eritrea

**Keywords:** Malaria vector control, Integrated vector management, Insecticide resistance management, Strategic planning, Operational frameworks, Vector surveillance, Community involvement, Monitoring and evaluation, Eritrea

## Abstract

**Background:**

Contemporary malaria vector control relies on the use of insecticide-based, indoor residual spraying (IRS) and long-lasting insecticidal nets (LLINs). However, malaria-endemic countries, including Eritrea, have struggled to effectively deploy these tools due technical and operational challenges, including the selection of insecticide resistance in malaria vectors. This manuscript outlines the processes undertaken in consolidating strategic planning and operational frameworks for vector control to expedite malaria elimination in Eritrea.

**Case description:**

The effort to strengthen strategic frameworks for vector control in Eritrea was the ‘case’ for this study. The integrated vector management (IVM) strategy was developed in 2010 but was not well executed, resulting in a rise in malaria transmission, prompting a process to redefine and relaunch the IVM strategy with integration of other vector borne diseases (VBDs) as the focus. The information sources for this study included all available data and accessible archived documentary records on malaria vector control in Eritrea. Structured literature searches of published, peer-reviewed sources using online, scientific, bibliographic databases, Google Scholar, PubMed and WHO, and a combination of search terms were utilized to gather data. The literature was reviewed and adapted to the local context and translated into the consolidated strategic framework.

**Discussion:**

In Eritrea, communities are grappling with the challenge of VBDs posing public health concerns, including malaria. The global fund financed the scale-up of IRS and LLIN programmes in 2014. Eritrea is transitioning towards malaria elimination and strategic frameworks for vector control have been consolidated by: developing an integrated vector management (IVM) strategy (2015–2019); updating IRS and larval source management (LSM) guidelines; developing training manuals for IRS and LSM; training of national staff in malaria entomology and vector control, including insecticide resistance monitoring techniques; initiating the global plan for insecticide resistance management; conducting needs’ assessments and developing standard operating procedure for insectaries; developing a guidance document on malaria vector control based on eco-epidemiological strata, a vector surveillance plan and harmonized mapping, data collection and reporting tools.

**Conclusion:**

Eritrea has successfully consolidated strategic frameworks for vector control. Rational decision-making remains critical to ensure that the interventions are effective and their choice is evidence-based, and to optimize the use of resources for vector control. Implementation of effective IVM requires proper collaboration and coordination, consistent technical and financial capacity and support to offer greater benefits.

## Background

Malaria remains an important vector-borne disease worldwide [1]. The global targets for malaria control and elimination are at least a 75 % reduction in malaria incidence and deaths by 2015 [2]. To achieve these goals, endemic countries implement vector control with indoor residual spraying (IRS), long-lasting insecticidal nets (LLINs) and larval source management (LSM) using environmental management and larviciding, alongside case management [3]. Vector control continues to be a critical component of contemporary malaria control efforts and is a vital attack weapon of malaria elimination. It is the only intervention capable of reducing transmission in the early stages of elimination [4]. Countries have deployed vector control tools with variable levels of coverage and success due to technical and operational constraints, including insecticide resistance in malaria vectors [5]. Integrated vector management (IVM), “a rational decision-making process for optimal use of resources for vector control”, is recommended as a platform for effective vector control [6] and the Global Plan for Insecticide Resistance Management (GPIRM) is an approach to mitigate selection and spread of resistance [7]. Endemic countries are also encouraged to develop guidelines for vector surveillance [8, 9]. To facilitate implementation, global operational plans for IVM and GPIRM have been developed [10, 11]. However, utilization of these approaches has been sub-optimal at country level.

Eritrea has been implementing vector control since the establishment of the National Malaria Control Programme (NMCP) in 1995. The number of malaria cases reported and treated by health facilities and community malaria agents increased from 141,213 in 1994, to 466,016 in 1998, and 293,671 in 1999 [12]. Historically, environmental management consisted of filling in or draining water from anopheline breeding sites, and larviciding of anopheline larvae-positive breeding sites with larvicide (temephos) or used motor oil. The use of *Bacillus thuringensis* var *israelensis* (Bti) or *Bacillus sphaericus* (Bs) was proposed for testing and deployment as and alternative to temephos. Distribution and use of LLINs began in 1995 in Gash-Barka and 1997 in Anseba. Biological control of mosquitoes using larvivorous fish, *Aphanius dispar*, a top-feeding predator of mosquito larvae and pupae, was evaluated on a small scale in both the Semenawi Keih Bahri and Southern Red Sea zobas. IRS was conducted in malaria epidemic-prone areas and in resettlement zones using DDT in Gash-Barka, Anseba and Dabub. Pyrethroids were proposed as a potential alternative insecticide to replace DDT. During 1998 and 1999, *Anopheles gambiae* were susceptible to 4 % DDT and 5 % malathion by standard World Health Organization (WHO) protocol in Gash-Barka, Debub and Anseba [12]. Notably, the Eritrean NMCP implemented an integrated vector control approach, not solely dependent on insecticides for the foreseeable future.

The WHO emphasizes IVM for combating vector-borne diseases (VBDs) [1]. The IVM approach has five key elements: advocacy; social mobilization and legislation; collaboration within the health sector and with other sectors; integrated approach; evidence-based decision making; and capacity building (Table 1). IVM salient attributes include: methods based on knowledge of factors influencing local vector biology; disease transmission and morbidity; use of a range of interventions, often in combination and synergistically; collaboration within the health sector and with other public and private sectors that impact on vectors; engagement of local communities and other stakeholders; and, a public health regulatory and legislative framework [1]. In Eritrea, *Anopheles arabiensis* is the primary malaria vector. *Anopheles d’thali, Anophelescinereus, Anophelesrhodesiensis, Anopheles squamosus*, and *Anopheles rupicolus* are secondary vectors [13]. However, diversity in adult behaviour and larval ecology preference among these species potentially impacts the ease with which each can be controlled. *Anopheles arabiensis* has a facultative indoor and/or outdoors resting behaviour and readily feed on either humans or cattle, making it more difficult to control. It prefers to breed in temporary, widely dispersed rain pools, hoof prints around the edges of dams. The other species have a predilection of feeding on animals and resting outdoors [13]. Like many other countries, Eritrea developed an IVM strategy in 2010 that focused on malaria [14]. The country has implemented a successful malaria control programme that integrates high LLIN coverage, focal IRS, targeted LSM using larviciding with temephos, early definitive diagnosis and timely case management, and effective information, education and behavioural change communication (IEC/BCC) [15].

**Table 1:** Key elements of an integrated vector management strategy [16]

No.	Element	Description
1.	Advocacy, social mobilization, and legislation	Promotion and embedding of IVM principles in designing policies in all relevant agencies, organizations and civil society; establishment or strengthening of regulatory and legislative controls for public health; empowerment of communities
2.	Collaboration within the health sector and with other sectors	Consideration of all options for collaboration within and between public and private sectors; application of the principles of subsidiarity in planning and decision making; strengthening channels of communication among policy-makers, VBD programme managers and other IVM partners
3.	Integrated approach	Ensure rational use of available resources by addressing several diseases, integrating non-chemical and chemical vector control methods and integrating with other disease control methods
4.	Evidence-based decision-making	Adaptation of strategies and interventions to local ecology, epidemiology and resources, guided by operational research and subject to routine monitoring and evaluation
5.	Capacity-building	Provision of the essential material infrastructure, financial resources and human resources at national and local level to manage IVM strategies on the basis of situation analysis

In Eritrea, malaria currently accounts for 2.1 % of the total outpatient morbidity and about 2.9 % of all hospital admissions. Approximately 70 % of the population resides in malaria-endemic areas, mostly in Gash-Barka, Debub, Semenawi Keih Bahri (SKB), and Anseba [15].Eritrea has demonstrated a consistent decline in malaria morbidity and mortality for the past 10 years [17]. The malaria burden declined from 110 cases/1000 population in 1998 to 11.9 cases/1000 population in 2012; malaria-specific deaths from 0.198 deaths/1000 population in 1998 to 0.0076 deaths/1000 population in 2012, and low nationwide *Plasmodium falciparum* prevalence. Nevertheless, incidence of malaria has risen in the last 3 years with an increase in malaria deaths from 2011 to 2012 coupled with occurrence of sporadic outbreaks in various sub-zones of the country [15]. Attempts to control the vectors have been intermittent and together with ecological factors, population movement and case management-related constraints, have had little apparent impact on the huge malaria burden in the period 2010–2014 [15]. The country has faced infrastructural and technical challenges, including insecticide resistance. Limited data on insecticide resistance profiles of vectors and their bionomics present constraints to effective and sustainable vector control.

During 2014 other VBDs emerged (dengue fever) and re-emerged (schistosomiasis and lymphatic filariasis) and malaria remains endemic and is increasing in four endemic regions of the country. This prompted a process to redefine and relaunch the IVM strategy with integration of other VBDs as the focus. Eritrea was successfully awarded the Global Fund to fight HIV/AIDS, tuberculosis and malaria rounds 2, 6 and 9 for malaria control in addition to previous World Bank grants under the HIV/AIDS, malaria, sexually transmitted diseases and tuberculosis (HAMSET) project, and was provided with seed funding by WHO to initiate the implementation of the GPIRM [15]. Cognizant of the need for an integrated approach to VBD control, the Ministry of Health (MoH) sought technical support from the WHO to conduct a thorough situation analysis of VBDs with the view to initiate studies in the context of IVM in Eritrea [15]. This paper outlines the processes that Eritrea undertook to consolidate strategic planning and operational frameworks for vector control, the challenges encountered, the lessons learnt from this experience, and how these lessons have informed vector control efforts in the country.

## Case description

The effort by the MoH and the WHO country office to strengthen strategic frameworks for vector control in Eritrea was the ‘case’ for this study. IVM strategy in Eritrea was developed in 2010 but was not well executed. The strategy was primarily used for malaria vector control. Until recently, the geographic distribution of most VBDs was not well assessed. Neither operational research nor sustainable vector control has been implemented for lymphatic filariasis and dengue due to lack of countrywide mapping. Insecticide resistance was selected in malaria vectors. Furthermore, systematic monitoring of vector bionomics and their resistance profiles, including a comprehensive entomological database, are lacking; available entomological laboratories are not functioning optimally; and, collaboration with relevant agencies has been very limited. Equally, IEC/BCC on VBDs, including improved environmental and personal hygiene, has been minimal. This situation resulted in a rise in transmission of malaria and other VBDs, prompting a process to redefine and relaunch the IVM framework with integration of other VBDs as the focus.

To revitalize IVM, Eritrea sought WHO technical support to develop and consolidate strategic frameworks for vector control by: updating the IVM Strategy (2015–2019), IRS and LSM guidelines; developing training manuals for IRS and LSM; training of national staff in malaria entomology and vector control, including insecticide resistance monitoring techniques and initiating the GPIRM; conducting needs’ assessments and developing standard operating procedure for insectaries; developing a guidance document on malaria vector control based on eco-epidemiological strata and harmonized mapping, data collection and reporting tools; elaborating an insecticide resistance monitoring and management plan and a vector surveillance plan.

Structured literature searches of published, peer-reviewed sources using online, scientific, bibliographic databases were utilized to gather data on VBDs in Eritrea. PubMed [18], Google Scholar [19] and the WHO Library [20] databases were searched without language restrictions for relevant articles published in the last decade with the search terms: (1) “malaria” and IVM; (2) NTDs and IVM; (3) NTDs and “vector control” and “prevention” and “surveillance”; (4) Eritrea; (1) and (4); (2) and (4); and, (3) and (4); vector control, epidemiology, malaria, visceral leishmaniasis (kala-azar), lymphatic filariasis (elephantiasis), schistosomiasis (Bilharzia), and dengue. Literature from Zambia, Namibia, Malawi, and South Sudan with similar framework strengthening approaches was also reviewed. Reference sections of all relevant located articles were also reviewed to identify more literature. Additional non-peer-reviewed documents in the MoH, such as annual reports and guidelines for vector control, were examined for information related to the subject. The literature was reviewed and applicable research findings and key concepts from Eritrea and other countries were discussed, adapted to the local context and translated into the consolidated strategic framework.

## Study area

**Fig. 1:**
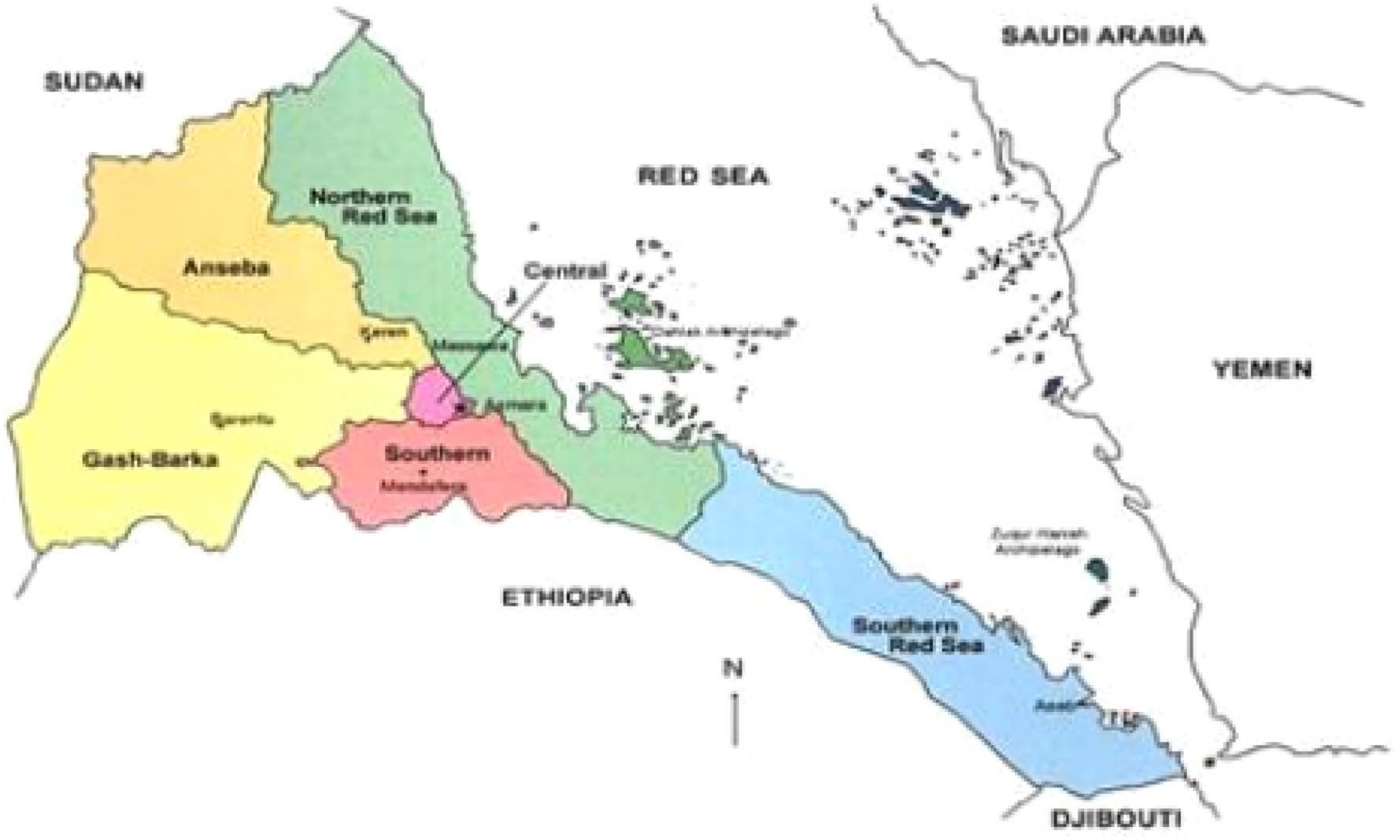
The Regional administration of Eritrea. Source [22]

Eritrea is located in the Horn of Africa, between latitudes 12°42′N and 18°2′N and longitudes 36°30′E to 43°20′E. It is bounded by the Sudan to the north and west, the Red Sea to the east, Ethiopia to the south and the Republic of Djibouti to the southeast (Fig. 1). The country has a surface area of 124,320 sq km and a topography characterized by highlands (2000 m.a.s.l.), lowlands (500–1000 m.a.s.l.) and coastal lands (500 m.a.s.l.). Administratively, the country is divided into six zones, 58 sub-zones and 699 administrative areas. By 2012, the population was estimated to be 3,952,788. Two rainy seasons during June–September (long) and October–March (short) with annual precipitation of 205–505 mm and average temperature spanning 16–50 °C. Notably, Eritrea is between latitudes 35°N and 35°S, the geographical range of VBDs globally [21].

## The status of vector-borne diseases in Eritrea

In Eritrea, five VBDs cause serious humanitarian and economic consequences. These include dengue fever, leishmaniasis, lymphatic filariasis, malaria, and schistosomiasis [23]. Malaria is highly seasonal, focal and unstable in the western lowlands and coastal plains and is prone to epidemics, with some perennial transmission along rivers, valleys, dams, as well as irrigation projects. The highlands are generally free from malaria, but are highly prone to malaria epidemics as a result of low immunity in these populations. The most common malaria parasite is *P. falciparum* which accounts for more than 84 % of all reported malaria cases and *Plasmodium vivax* is responsible for the remaining 16 % [24]. VBDs exhibit varying levels of endemicity and overlap with malaria in all the six zones (Tables 1, 2). Schistosomiasis distribution is influenced by altitude with prevalence >25 % occurring at altitude 1220–2380 m [14]. Lymphatic filariasis has very low prevalence in Eritrea. *Anopheles arabiensis*, the main vector of malaria may together with culicines contribute to lymphatic filariasis transmission. In recent years, dengue fever has rapidly spread to almost the entire country [25]. There is a need to map VBD distribution in order to guide implementation of mass drug administration for neglected tropical diseases (NTDs) and vector control.

**Table 2:** Co-endemicity of vector-borne diseases in Eritrea by zone [26]

Zone	Malaria	Dengue fever	Leishmaniasis	Lymphatic filariasis	Schistosomiasis	Trachoma
SRS	+	+	+	+	+	+
SKB	+	+	+	+	+	+
Anseba	+	+	+	+	+	+
G-Barka	+	+	+	+	+	+
Debub	+	+	+	+	+	+
Makael	+	+	+	+		+

## Policy and guidance for vector-borne disease control in Eritrea

By definition, IVM is “a rational decision-making process for optimal use of resources for vector control”. An IVM policy considers the existing health infrastructure and resources, employs a multi-disease approach, integrates all accessible and efficacious chemical, biological or environmental interventions, encompasses other sectors and communities, and strives to reinforce vector control management systems [1]. Accordingly, an IVM-based process should be cost-effective, guided by operational research and subject to routine monitoring and evaluation of impact on vector populations and disease transmission, including development of an infrastructure, financial resources and adequate human resources to manage and implement integrated vector control programmes at national and local levels [1]. Eritrea implemented malaria vector control with the establishment of the NMCP in 1995. There has since been active participation of communities in vector control activities as a result of intensive health promotional campaigns and provision of necessary materials. Considering the endemicity overlaps and transmission similarities of some VBDs (Tables 2, 3), an integrated approach in controlling the diseases is recommended wherever feasible in order to best achieve set goals. This requires adherence to all the five key strategic elements of the approach. Recognizing that Eritrea has implemented IVM over the years, the most useful and feasible options for IVM to be applied to the framework are: capacity building, integration across diseases, different sectors and communities, evidence-based approach, and collaboration within the health sector and other sectors [6] (Table 1). In 2010 the IVM approach was adopted and implemented as the main platform for vector control and included deployment of IRS and LLINs supplemented by LSM [14]. However, due to increasing malaria transmission and challenges in vector control, the country set out to update, revise and widen the scope of its IVM strategy, which is documented in the following sections.

**Table 3:** The burden of emerging and re-emerging vector-borne diseases endemic in Eritrea

Disease	Causative agent	Vector	Distribution	Burden	Intervention	Ref
Malaria	*P. falciparum*	*An. gambiae,* *An. arabiensis,* *An. funestus*	Countrywide	1.1 % prevalence	ITNs, IRS, LSM	[26–28]
Lymphatic filariasis (Elephantiasis)	*Wuchereria bancrofti*	*An. gambiae,* *An. arabiensis,* *An. funestus*	Countrywide	Very low prevalence	Large-scale distribution of LLINs for vector control	[26, 29]
Schistosomiasis (Bilharzia)	*Schistosoma haematobium* *Schistosoma mansoni*	*Bulinus* and *Biomphalaria spp*	Gash-Barka, Debub, Anseba, Maekel	2.5 % prevalence (0–100 %)	Molluscicides	[26, 29]
Dengue	Dengue virus	*Aedes aegypti, Aedes albopictus*	Gash Barka, SKB, Southern Red Sea (SRS), Debub, Anseba, and Maekel	88 % positivity rate (sub serotype-1)	Increased surveillance, LSM	[24, 26]
Trachoma	*Clostridium Trachomatis*	*Musica sorbens* (Bazaar fly)	Countrywide	–	Environmental improvement	[26]
Visceral leishmaniasis (Kala-azar)	*Leishmania donovani*	*Phlebotomus orientalis* *Phlebotomus* *Martini* and *Phlebotomus celiae*	Gash Barka, SKB, Debub, Anseba, and Maekel	–	LLINs in highly endemic communities	[26]

## Guidelines for integrated vector management from 2010 to 2014

The MoH’s determination to integrate control of all VBDs occurring in Eritrea was demonstrated by the development of IVM guidelines in 2010 [14]. The guidelines were consistent with the National Malaria Strategic Plan (NMSP 2010–2014), which aimed to scale up coverage of transmission-reducing tools to control and eliminate malaria [22] and the Strategic Plan of Action for Integrated Control of NTDs (2010–2014), which sought to integrate the control of all NTDs occurring in Eritrea [30]. The guidelines were also in line with WHO recommendation that countries adopt an IVM approach for the control or elimination of VBDs [10]. The national IVM guidelines were developed through extensive consultations with Ministry of Agriculture and Ministry of Lands, Water and Environment with expert technical guidance from WHO. The intention was to extend IVM implementation to control other VBDs utilizing a range of evidence-based interventions that include environmental management, the safe and judicious use of insecticides in IRS, and LLINs and larviciding/mollusciciding. The objective of the IVM guidelines was to give direction and guidance on integrating VBD control with the NMCP in Eritrea.

## Vector control interventions

Vector control is conducted by the MoH with oversight of the Communicable Diseases Control Division. The control of schistosomisis, dengue, leishmaniasis, and lymphatic filariasisis is the mandate of the Neglected Tropical Diseases Unit. Malaria vector control falls under the NMCP. Community mobilization has been critical in vector control. Community health agents are involved in LLIN distribution, and larvicide applicators as well as spray operators for IRS are also drawn from communities. Vector control using selected IRS, distribution of LLINs and targeted LSM has been deployed in the context of IVM with varying degrees of coverage and success [31]. The use of LLINs is the main vector control strategy in Eritrea. The programme aims to attain universal coverage by providing one LLIN for every two people at risk, and ensuring 80 % usage by 2019. LLINs are distributed through routine, free distribution via antenatal clinics to pregnant women and newborn children and periodic free mass distribution (every 3–4 years). Tax exemption of importation of malaria control commodities has been effected in Eritrea. IRS was the primary malaria vector control intervention prior to the introduction of LLINs. Intradomiciliary spraying is conducted annually prior to the rainy season in selected highly malarious areas in Gash-Barka and Debub zones to prevent epidemics and to reduce transmission. Since 1998, IRS using dichloro-diphenyl-trichloro-ethane (DDT) or malathion was done routinely. Data for IRS are collected according to WHO-recommended format with spraying forms capturing the number of villages covered, number of structures sprayed, population covered, and amount of insecticide used [26]. In 2010, guidelines for IRS were developed for implementation of the intervention [32]. LSM, including simple environmental management through draining and burying ditches and larviciding using temephos and *Bti* is implemented as a supplementary intervention. Community engagement and participation has been critical for successful LSM as larvicide applicators are drawn from the communities [15]. Entomological monitoring and resistance surveillance have been inadequately conducted to guide vector control. Monthly mosquito collections and annual susceptibility tests are conducted on only a small scale in Tesseney sub-zoba in Gash-Barka region.

## Vector control spatial scale, performance and policy changes

By 2015, approximately 1.8 million LLINs were distributed since 2008. Between 2008 and 2012, coverage of at least one LLIN per household increased from 70.9 to 88.5 % [31]. Accordingly, usage of LLINs (people who slept under an ITN the previous night) increased from 48.9 to 67.4 % among children under 5 years old and from 47.6 to 60.1 % in women aged 14–49 years [31]. Administrative universal coverage (the number of nets distributed expressed as percentages of the target number needed to satisfy the goal of at least one net in 80 % of households) was 76 %. Sprayed structures ranged from 75,019 in 2008 to 290,978 in 2013 with average operational coverage of 88.4 and 92.3 %, protecting 251,641 and 278,857 people, respectively. However, in 2009 and 2010 the operational coverage of IRS in Gash-Barka reduced due to targeting of epidemic-prone areas and procurement challenges [15]. In 2012, the coverage of IRS was 49.4 and 31.5 % in Gash-Barka and Debub, respectively [30]. Between 2008 and 2013, a total of 27,588 and 47,347 breeding sites were filled and larvicided, respectively. The number of sites filled increased from 27,339 in 2008 to 32,962 in 2013. Total breeding sites treated with larvicides decreased from 81,865 in 2008 to 64,352 in 2013. Quantities of temephos applied decreased from 363,110 in 2008 to 224,352 in 2013 [15]. The number of recorded malaria cases increased from 25,969 in 2008 to 34,678 in 2013.

Entomological studies have shown high *An. arabiensis* densities in Gash-Barka and Anseba regions, necessitating heightened vector control efforts [13]. From 2009 to 2014, insecticide resistance surveillance conducted in Gash-Barka detected high resistance levels to 4 % DDT (67.3 % mortality), 0.75 % permethrin (60.9 %) and 0.05 % lambda cyhalothrin (61.9 %) with suspected resistance to 0.05 % deltamethrin (91.3 %) and complete susceptibility to 5 % malathion (98.6 %), pirimiphos methyl (100 %) and 0.1 % bendiocarb (100 %) [15]. These findings have implications for insecticide-based vector control. The efficacy of insecticides on LLINs and IRS has been evaluated in 2009 and 2012 [29]. The mean mortality on LLINs used for 2–4 years dropped from 88.6 to 43.1 %. Similarly, the residual efficacy of insecticides on sprayed surfaces showed mean mortality rates of 92.6 % on mud walls and 93.6 % on painted walls compared to 80 % on brick walls [32]. In 2012, selection of insecticide resistance in malaria vectors prompted a policy shift from DDT to a rotation of pyrethroids and carbamates.

## Challenges for vector control

Vector control in Eritrea has encountered several risks and challenges (Table 4). While IVM guidelines were intended to integrate all VBDs in the country, they have been used primarily for malaria vector control. Until recently, the geographic distribution of most VBDs was not well assessed in Eritrea [33]. Neither operational research nor sustainable vector control has been implemented for lymphatic filariasis and dengue due to lack of comprehensive countrywide mapping. The guidelines had a strong emphasis on IRS with limited consideration of other vector control tools and were not fully utilized due to operational and technical capacity constraints. There are no entomologists to guide IVM activities at national, zonal or sub-zone levels. Selection of insecticide resistance in malaria vectors is threatening to compromise the success of the programme. Regular and systematic monitoring of vector bionomics and insecticide resistance and a comprehensive entomological database are lacking. While field entomological laboratories that are meant to facilitate this effort exist in three malarious zones, insectaries have not been sufficiently supported in terms of human resources, commodities and equipment to optimize their functionality [34]. Effective transmission interruption through comprehensive vector control and collaboration with relevant agencies has been very limited. There is a lack of technically competent local stakeholders for decision-making, with no relationships with external research institutions for research support and experience sharing. The absence of dedicated transport for entomology and vector control activities is a persistent constraint. Equally, IEC/BCC on VBDs, including improved environmental and personal hygiene, has been minimal. Accordingly, these challenges revitalized the rewriting and updating the IVM strategy.

**Table 4:** Challenges and risks encountered in vector control and recommendations for improvement in Eritrea

Challenges and risks encountered	Recommendations for improvement
Limited requisite infrastructure, technical and institutional capacity for entomology laboratory	Strengthen the infrastructure (sentinel sites, laboratory and insectary facilities), technical and human resource capacity for entomology at established sentinel sites
Collaboration with external research and academic institutions to support decision making for IRM and vector control is non-existent	Strengthen collaboration of all partners with vested interest in entomological and insecticide resistance monitoring including internal and external research and academic institutions
Lack of a database for vector control and key entomological parameters, including insecticide resistance with only very minimal skills for data management	Establish data management systems and operate a comprehensive entomological database at national level
Limited adherence to existing guidelines for vector control	Regularly update the IVM guideline, to encompass all strategic aspects of IRS, LLINs and LSM, according to prevailing eco-epidemiological stratification of malaria in the country
Very minimal vector surveillance has been conducted due to limited technical capacity. Limited capacity for IRS quality monitoring and lack of consistency in conducting this activity	Improve technical capacity for vector surveillance and select and implement Interventions based on spatial and temporal distribution of the vectors species, including resistance profiles and heterogeneities in their resting and feeding attributes
Limited resistance data, including underlying mechanisms due to minimal technical capacity resulting in ill-informed decisions on insecticide use for vector control	Implement the GPIRM by regularly revising and elaborating insecticide resistance monitoring and management plan guided by vector surveillance
Lack of IRS supervision checklists for monitoring spraying activities	There is need to develop LLINs and LSM guidelines to streamline quantification and distribution of commodities, and IRS supervision checklists
There is weak coordination of partner involvement and contribution in LLIN distribution	Coordination of partners involved in LLIN distribution should be strengthened and the contribution of each partner should be well documented

## Opportunities for vector control in Eritrea

Despite the constraints that the country has faced, opportunities for streamlining vector control exist in Eritrea. The availability of funding from the Global Funds to fight HIV/AIDS, Tuberculosis and Malaria for establishing a training programme in Malaria Entomology and Vector Control with local training institutes, particularly the Asmara College of Heath Sciences, is timely. The existence of infrastructure such as insectaries and entomology laboratories in three malarious zones provides prospects for enhanced entomological monitoring and surveillance. Training of MoH personnel, particularly public health officers, from zobas and sub-zobas in malaria entomology and vector control will increase the number of personnel with much-needed competence. The elaboration of the IVM strategy and development of guidelines and training manuals for IRS and LSM, coupled with the availability of standard operation procedures for sentinel sites, including entomology laboratory and insectary activities, provides a platform for effective vector control. The development of an insecticide resistance monitoring and management plan and a vector surveillance plan will further augment vector control efforts. Collaboration with Asmara College of Health Sciences and available technical support from WHO/AFRO provides hope for evidence-based and efficacious deployment of interventions. In 2014, NTD mapping was conducted in Eritrea and provides latest data on scale of lymphatic filariasis and schistosomiasis that is critical for an integrated approach [33].

## Consolidating strategic frameworks for vector control

In Eritrea malaria is a public health concern, with transmission slightly exacerbated by poor vector control, selection of insecticides resistance in malaria vectors and insufficient technical resources. Strategic planning and operational frameworks for vector control were consolidated to maintain the intervention to lower transmission and to accelerate transition towards malaria elimination. Eritrea has embarked on efforts to update the IVM strategy and streamline vector control in response to current prevailing, dynamic eco-epidemiology of malaria and other VBDs, and to respond to the threat of insecticide resistance by elaborating a rational IRM plan and integration of other VBDs. Considering the challenges faced in the past and existing opportunities for contemporary vector control, the MoH sought for technical assistance from World Health Organization African Regional Office (WHO/AFRO) and The Eritrean WHO Country Office (WHO/ERI) to facilitate implementation of key strategic framework activities, including training of national staff in insecticide resistance monitoring techniques and initiating the GPIRM process following WHO’s guidelines; providing technical support in conducting VBD-related studies; giving guidance on malaria vector control based on eco-epidemiological strata, capacity building in malaria entomology and vector control and harmonizing mapping, data collection and reporting tools.

## The IVM Strategy for the period 2015 to 2019

In 2014, the current IVM Strategy (2015–2019) for Eritrea was developed with full consideration of the prevailing epidemiological situation in the country and the currently existing opportunities for vector control [23]. The strategy has been greatly informed by the challenges faced during the life span of the preceding guideline [14]. The strategic frameworks have been formulated to support the NMSP (2015–2019) [34] and the National Master Plan for NTDs (2015–2020) [35] and also to strengthen efforts aimed at integrating VBDs present in the country based on IVM principles [6]. The IVM strategy (2015–2019) differs from the previous one in that the process of updating was backed by comprehensive situation analysis of profiles for VBDs that could potentially be combined in their control/elimination (Table 5), extensive review of IVM guideline (2010–2014) and alignment of vector control to the current eco-epidemiology of malaria. The objectives and activities to be implemented, including programme indicators, are clearly stipulated for all key interventions. The Strategy incorporates components on monitoring and evaluation and operations research, IRM, programme management, an implementation plan, a performance framework, and a budget indicating funding with estimated implementation costs [21]. To effectively control VBDs, the implementation process for the Strategy envisions strong adherence to the five key elements of the IVM approach (Table 4) and national policy [36]. In this regard, priority strategic framework activities have been aligned with the current IVM strategic plan as outlined in the next section.

**Table 5:** Integrated vector management components and the disease vectors they control in Eritrea

Component	Intervention	Vector targets	Vector-borne diseases
Chemical control	LLINs	*Anopheles, Culex, Aedes, Phlebotomus* sp.	Malaria, leishmaniasis, lymphatic filariasis, dengue
	IRS	*Anopheles, Culex, Aedes, Phlebotomus* sp.	Malaria, leishmaniasis, lymphatic filariasis, dengue
	Larviciding	*Anopheles, Culex, Aedes, Phlebotomus, Biomphalaria* sp.	Malaria, leishmaniasis, lymphatic filariasis, dengue, schistosomiasis
	Space spraying	*Anopheles, Culex, Aedes, Phlebotomus* sp.	Malaria, leishmaniasis, lymphatic filariasis, dengue
	Household products	*Anopheles, Culex, Aedes, Phlebotomus*	Malaria, leishmaniasis, lymphatic filariasis, dengue
Environmental management and sanitation	Environmental manipulation and modification	*Anopheles, Culex, Aedes, Phlebotomus, Biomphalaria* sp.	Malaria, leishmaniasis, lymphatic filariasis, dengue, schistosomiasis
Biological control	Larval control	*Anopheles, Culex, Aedes, Phlebotomus* sp.	Malaria, leishmaniasis, lymphatic filariasis, dengue, schistosomiasis
	Predators and competitors	*Anopheles, Culex, Aedes, Phlebotomus, Biomphalaria* sp.	Malaria, leishmaniasis, lymphatic filariasis, dengue, schistosomiasis

## Priority strategic framework activities

### Insecticide resistance monitoring and management

Eritrea has embarked on efforts to implement the GPIRM process according to the WHO guidelines [7]. However, operating the GPIRM requires elaboration of an IRM plan subject to regular revision and updating. Following the urgent need for updated information on insecticide resistance profiles of vectors for informed decision-making, training to build competence on how to set up and run a routine system on resistance monitoring was conducted in 2014 [37]. A protocol for routine monitoring of insecticide resistance in malaria vectors was developed [38] based on standard WHO procedures [39]. This was to facilitate monitoring of spatiotemporal insecticide resistance status of the malaria vector in different malaria epidemiological settings of the country. A system for routine insecticide resistance surveillance and monitoring has been established in three highly malarious zones of Eritrea: Gash-Barka, Debub and Anseba. Insecticide resistance in malaria vectors will be monitored over time in multiple sentinel sites. The core elements of the IRM plan are consistent with guidelines developed by the WHO Global Malaria Programme and the roll back malaria (RBM) vector control working group [7, 11]. However, the IRM plan should consider and incorporate resistance profiles of vectors for other diseases.

### Guidance on malaria vector control based on eco-epidemiological strata

The NMCP in Eritrea has developed a guidance document for vector control interventions based on the prevailing malaria eco-epidemiological strata and socio-economic factors in the country [40] to provide approaches to evidence-based deployment of appropriate vector control interventions in the context of IVM in different settings. The guidance document outlines vector control in different epidemiological strata, based on the concept of stratification of the six main global eco-epidemiological types, indicative of particular vector bionomics, transmission status and appropriate vector control measures [40]. In Eritrea, this includes: the epidemic-prone highland and desert fringes, forest and forest fringes in the Southern Red Sea zone, coastal areas and wetlands in Semenawi Keih Bahri one, urban and peri-urban areas of major towns and agricultural development projects in Gash-Barka and Debub [27]. The IVM strategy is supported by a compendium of updated guidelines and training manuals for LSM [28] and IRS [41]. The guidelines have been aligned with the current WHO operational manuals and recommendations [42, 43]. To effectively implement these interventions, a vector surveillance plan is being developed.

### Capacity building in malaria entomology and vector control

Limited data on insecticide resistance profiles of vectors and their bionomics present invariable constraints to effective and sustainable scaled-up malaria vector control. Strong entomological teams at national and local levels are crucial to coordinate routine monitoring of resistance, data analysis and interpretation to inform policy decisions, translate policies and guidance into action. Strengthening and streamlining vector control efforts in response to the current eco-epidemiology of malaria and the challenge of insecticide resistance is needed. The country has embarked on capacity building in malaria entomology and vector control for public health officers in the MoH to support and provide expertise for evidence-based, operational implementation of the IVM strategy. To strengthen malaria vector control, entomological laboratories have been established and entomological monitoring sentinel sites set up in three highly malarious zones. Initial training of national staff in entomological monitoring and insecticide resistance surveillance was conducted in 2014 [44]. Furthermore, an international training course on malaria planning and management has been conducted [45]. Job descriptions and standard operating procedures (SOPs) for insectaries have been developed [46]. Further building of capacity and systems for timely entomological, insecticide resistance and epidemiological monitoring across a network of sentinel sites to assess the impact of resistance mechanisms will be crucial.

### Harmonizing mapping, data collection and reporting tools

Considering the co-endemicities of different VBDs (schistosomiasis, lymphatic filariasis, dengue, malaria and visceral leishmaniasis), efforts have been embarked upon to harmonize mapping, data collection and reporting tools. As the country re-orients towards malaria elimination, it is imperative that data collection is streamlined within the NMCP. In 2014, national data on the implementation on malaria vector control, including coverage of IRS and status of ownership and use of LLINs during the period 2008–2013, were collated [31]. Selecting and implementing interventions based on vector species composition, spatial and temporal distribution, including their insecticide resistance profiles and heterogeneities in resting and feeding attributes, will be needed. The programme has developed a vector surveillance plan to provide evidence for decision-making in IVM, for assessing the programme’s impact on vector populations and for monitoring and evaluation where the surveillance sites are located in or near the implementation settings. This is likely to be achieved given the country’s effort to build capacity of public health officers in the MoH in malaria entomology and vector control. Establishment of a comprehensive entomological database at national level has also been prioritized to optimize decision-making. To circumvent constraints regarding planning, targeting, operations, logistics, advocacy, and monitoring and evaluation for IRS, the NMCP plans to conduct a thorough and timely geographical reconnaissance supported by GIS-based satellite imagery.

### Consolidating collaboration and coordination

Mobilization of requisite human and financial resources would require establishing intersectoral collaboration for decision-making in entomology and vector control. In this regard, linkage with external research and teaching institutions, including collaboration with local training institutes, such as Asmara College of Heath Sciences, is being launched. Such collaboration with external research institutions, with development of a database and establishing modalities for sharing and disseminating information, will facilitate determination of underlying resistance mechanisms that is vital for rational IRM strategies and to inform policy formulation. The implementation of these activities will be coordinated by the NMCP.

## Discussion

Through implementation of effective interventions based on the IVM policy [14], the malaria control programme in Eritrea has become successful in certain areas. It has now detected some failures and/or difficulties with its present operations and techniques (Table 1). Consequently, malaria cases have increased since 2012 and other VBDs are emerging and re-emerging. This situation could provide lessons for other VBD-endemic countries. As Eritrea consolidates malaria pre-elimination and transitions towards elimination, there is renewed interest in vector control resulting in both strengthening of strategic frameworks and expansion of IRS and distribution of LLINs, including an attempt to integrate other VBDs.

Poor targeting of vector control interventions has adversely affected their efficacy in earmarked areas due to lack of harmonization in timing of combination of interventions and areas targeted [40]. Different tools have been targeted at specific locations with high malaria transmission. In Gash-Barka and Debub, IRS and LLINs are co-implemented and supplemented with LSM. In Anseba, distribution of LLINs is the only intervention supplemented by LSM. In Semenawi Keih Bahri zone, nomadic populations and lack of permanent housing structures ideal for IRS preclude effective vector control [40]. With no empirical studies conducted to inform decisions, implementation has been characterized by technical and operational disparities. The need for operational research to generate local entomological and epidemiological evidence is notable. While over two million LLINs have been distributed to achieve universal coverage, thorough needs’ assessments and revised LLIN guidelines will be required. LSM implementation by untrained larvicide applicators has been ad hoc and without technical guidance. LSM should be deployed according to national guidelines [28]. Spray operators have received insufficient 3-day training and are unable to acquire the necessary skills and competence in spraying techniques. This may have implications for supervision, monitoring and evaluation of spraying activities and could potentially compromise the quality of the programme. In Gash-Barka, the IRS programme lacks camp sites, requiring spray operators having to commute long distances to operational areas. IRS should be deployed according to the national guidelines [41]. All these challenges could have potentially contributed to the observed increase in malaria cases in the last 3 years in Eritrea.

Insecticide susceptibility tests on malaria vectors and contact bioassays on both sprayed walls and bed nets have been intermittently conducted in Eritrea. This information is critical for planning purposes and should be collected regularly and professionally [32]. Selection of resistance in major malaria vectors and the lack of updated entomological data in operational settings has implications for the control programme [15]. Insecticide resistance could potentially jeopardize the long-term benefit of existing and newly developed insecticides, and it can elicit control failure as has already been seen in South Africa, and constrain insecticide choices [46, 47]. Although detecting resistance in laboratory tests does not necessarily mean that field applications are useless, Eritrea has embarked on efforts to respond to the threat of this phenomenon and the dynamic malaria landscape by conducting a comprehensive vector control analysis and updating pertinent strategic documents in line with the current eco-epidemiology of malaria. The choice and operations of vector control tools should consider vector species composition, spatial and temporal distribution, bionomics, including resistance profiles and adoption of resistance management tactics. Eritrea has enhanced resistance monitoring to inform selection of insecticides for vector control [37]. The country has developed specific SOPs to guide vector surveillance and data collection for real-time decision making to improve vector control in the context of IVM.

The recent mapping of lymphatic filariasis and schistosomiasis [33] coupled with a review of dengue outbreaks from 2005 to 2015 provides updated information to facilitate their integrated control in Eritrea. The presence of a single major vector species for malaria in Eritrea makes targeting of vector control interventions easier than to multiple vectorial systems with variable behaviour in different ecotypes [13]. However, an integrated approach would require strengthening infrastructural, technical and institutional capacities for effective vector control for malaria and other VBDs and building systems for rigorous entomological, resistance and epidemiological monitoring and surveillance to ensure the successful of evidence-based present and future interventions. Evidence-based decision making and technical skills capacity building will be critical for effective IVM [7]. This necessitates designation of an entomologist to oversee and guide deployment of interventions as well as personnel for specific interventions, i.e., IRS, LLINs and LSM. Equally, different studies need to be conducted to assess the need and amenability of the interventions, including determining knowledge, attitudes and practices (KAP) on misuse/abuse of nets for carrying hay and other items and ascertaining the contribution of imported cases.

Well-established IVM programmes with adherence to the five key attributes demonstrate enhanced intervention impact and leverage additional resources [3]. Accordingly, with strengthened strategic frameworks, Eritrea is likely to accelerate progress towards malaria elimination with concomitant impact on other VBDs. However, resistance has developed in malaria vectors in neighbouring countries, such as Ethiopia [48] and Sudan [16]. Eritrea, Bioko, Equatorial Guinea, Namibia and Zambia are among the few nations that have developed IRM plans to prevent development and spread of insecticide resistance and have trained local staff in entomological and resistance monitoring [16, 49]. Eritrea should establish regional collaboration networks with international research and academic institutions for development, harmonization and coordination of methodologies and decision-making [50]. To this effect, creating an advisory body to recommend policy changes and adjustments in vector control technical options on the basis of evidence generated by surveillance will be required. Eritrea needs to strengthen capacities to evaluate and introduce new vector control tools and application technologies [50], including adopting contemporary innovative strategies such as durable wall linings, attractive toxic sugar baits, long-lasting topical repellants, spatial repellents, entomopathogenic bacteria traps, fungus-impregnated targets, eave tubes [51], and new molecules for IRS, i.e., chlorfenapyr [52] plus improved housing [53], drainage and space spraying [54]. Effective and sustained vector control requires well-coordinated IEC/BCC to promote awareness, compliance and ownership of interventions, coupled with unremitting advocacy for political and local financial support [55].

## Conclusion

Eritrea has successfully consolidated strategic frameworks for malaria vector control. Rational decision-making remains critical to ensure that interventions are effective and their choice is evidence-based to optimize the use of resources. Implementation of an effective IVM strategy requires proper collaboration, advocacy and consistent technical and financial capacity and support. An insecticide resistance monitoring and management plan involving a decision-making body would be essential for effective and sustainable insecticide-based vector control. A well-coordinated IVM approach supported by viable vector surveillance may offer greater benefits.

## Abbreviations

Bti: *Bacillus thuringensis* var *israelensis*
DDT: Dichloro-dimethyl-trichloroethane
GPIRM: Global plan for insecticide resistance management
HAMSET: HIV/AIDS, malaria, sexually transmitted diseases and tuberculosis
IEC/BCC: Information, education and behaviour change communication
IRS: Indoor residual spraying
KAP: Knowledge, attitudes and practices
LLINs: Long-lasting insecticidal nets
IRM: Insecticide resistance management
LSM: Larval source management
IVM: Integrated vector management
MoH: Ministry of Health
NMCP: National Malaria Control Programme
VBDs: Vector-borne diseases
NTDs: Neglected tropical diseases
RBM: Roll back malaria
SOP: Standard operating procedures
WHO: World Health Organization
